# Supplementary material for: Partially coherent radar unties range resolution from bandwidth limitations
Source: Nat Commun. 2019 Mar 29;10:1423. doi: 10.1038/s41467-019-09380-x (PMC6441069; doi:10.1038/s41467-019-09380-x)
Supplement: Supplementary file 1 — Supplementary Information [file 41467_2019_9380_MOESM1_ESM.docx]

**Supplementary Note 1: Details for the derivation of equations 3 and 5**

Substituting Eq.1 into Eq.2 (without taking the expectation value and ignoring the noise term) and considering a constant coherence length with index *m*, leads to the following expression:

| $C_{m}^{\mathrm{SST}}=\frac{A}{N\tau_{m}}\int_{T_{m}}^{T_{m}+N\tau_{m}} \mathrm{Cos}\left( \omega t+\sum_{n=0}^{N-1} \mathrm{rect}\left( \frac{t-n\tau_{m}-T_{m}}{\tau_{m}} \right)\varphi_{nm} \right)\mathrm{Cos}\left( \omega\left( t-\tau\right)+\sum_{q=0}^{N-1} \mathrm{rect}\left( \frac{t-\tau-q\tau_{m}-T_{m}}{\tau_{m}} \right)\varphi_{qm} \right)dt .$ | (A1) |
| --- | --- |

Using the trigonometric identity $Cos\left( a \right)Cos\left( b \right)=\frac{1}{2}\left( Cos\left( a+b \right)+Cos\left( a-b \right) \right)$ and the fact the integration domain is much larger than the period of the carrier, the remaining term is

| $C_{m}^{\mathrm{SST}}\approx\frac{A}{2N\tau_{m}}\int_{T_{m}}^{T_{m}+N\tau_{m}} \mathrm{Cos}\left( \omega\tau+\sum_{n=0}^{N-1} rect\left( \frac{t-n\tau_{m}-T_{m}}{\tau_{m}} \right)\varphi_{nm}-\sum_{q=0}^{N-1} \mathrm{rect}\left( \frac{t-\tau-q\tau_{m}-T_{m}}{\tau_{m}} \right)\varphi_{qm} \right)dt .$ | (A2) |
| --- | --- |

The integration domain may be transformed by substituting $t\to t-T_{m}$. During constant coherence times of duration $\tau_{m}$, there are two regions of the cosine. The first is in the domain ($0,\tau)$, where $\varphi_{nm}\neq\varphi_{qm}$ (transmitted phase is not the same as the returning phase), the second is in the domain ($\tau,\tau_{m})$ where $\varphi_{nm}=\varphi_{qm}$. Since this is true for all n (all constant coherence intervals), the sum may be taken out of the integral, integrating separately over constant coherence intervals of the transmitted signal. The integral may then be split into two parts according to the two domains mentioned earlier:

| $C_{m}^{\mathrm{SST}}=\frac{A}{2N\tau_{m}}\sum_{n=0}^{N-1} \left( \int_{n\tau_{m}}^{n\tau_{m}+\tau} \mathrm{Cos}\left( \varphi_{n,m}-\varphi_{n-1,m}+\omega\tau\right)dt+\int_{n\tau_{m}+\tau}^{\left( n+1 \right)\tau_{m}} \mathrm{Cos}\left( \omega\tau\right)dt \right) .$ | (A3) |
| --- | --- |

Now, the expectation value and variance may be taken in order to obtain Eq.3 and 5.
